# Torsional component of microsaccades during fixation and quick phases during optokinetic stimulation

**DOI:** 10.16910/jemr.13.5.5

**Published:** 2020-10-20

**Authors:** Shirin Sadeghpour, Jorge Otero-Millan

**Affiliations:** Johns Hopkins University, Baltimore, MD, USA; University of California Berkeley. Berkeley, CA, USA

**Keywords:** Eye movement, eye tracking, saccades, microsaccades, torsion, fixation, optokinetic stimulation

## Abstract

While many studies have characterized the eye movements during visual fixation, includ-ing microsaccades, in most cases only horizontal and vertical components have been rec-orded and analyzed. Thus, little is known about the torsional component of microsaccades. We took advantage of a newly developed software and hardware to record eye movements around the three axes of rotation during fixation and torsional optokinetic stimulus. We found that the average amplitude of the torsional component of microsaccades during fixation was 0.34 ± 0.07 degrees with velocities following a main sequence with a slope comparable to the horizontal and vertical components. We also found the size of the tor-sional displacement during microsaccades was correlated with the horizontal but not the vertical component. In the presence of an optokinetic stimulus a nystagmus was induced producing a more frequent and larger torsional quick phases compared to microsaccades produced during fixation with a stationary stimulus. The torsional component and the vertical vergence component of quick phases grew larger with higher velocities. Addition-ally, our results validate and show the feasibility of recording torsional eye movements using video eye tracking in a desktop mounted setup.

## Introduction

**Figure 1.**
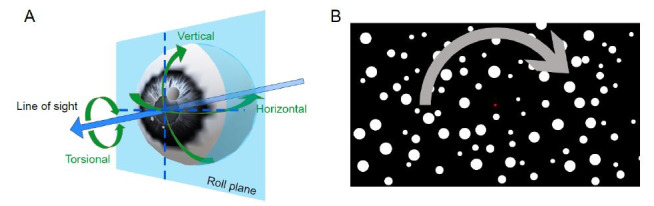
A) The three axes of rotation of the eye. B) Approximate display used during the experiment.

Our eyes are never entirely still. Even when attempting to keep our gaze fixed on a small stationary target, fixational eye movements continuously occur (1, 2). Fixational eye movements include microsaccades, drift, and tremor. Microsaccades are saccade-like movements typically less than 1 degree that occur once or twice per second. Drift is a slow movement that occurs in between microsaccades, and tremor is a fast and small oscillatory movement that cannot be measured with the most commonly used eye trackers (3). Most recent studies record eye movements using video eye trackers. Unlike a scleral search coil, video eye tracking does not interfere with and distort saccade dynamics (4). These devices typically use image processing techniques to find the pupil and track it as it moves. Then, the degrees of rotation of the eye in the horizontal and vertical dimensions can be calculated using a calibration. However, the eye does not move only in two dimensions; it can also rotate around the line of sight (torsional dimension, see Figure 1A). Torsional eye movements do not change the position of the pupilupil's position in the video image and cannot be recorded with most devices that rely on pupil tracking. Multiple methods have been developed to use video signals to measure torsion, but their use is not widespread, mainly because most of them require off-line tracking (5, 6) to extract and visualize torsional eye position. Other non-video-based methods such as the scleral search coil method have been used in the past to measure eye movements around three axes of rotation (7).

Previous studies have measured the stability of torsion during fixation and how it compares to the horizontal and vertical components (8, 9). They found that the instability of the torsional component was the largest of the three. Van Rijn and colleagues focused on studying the differences between the control of cycloversion and cyclovergence (7, 9, 10), that is, torsional movements in which both eyes rotate in the same direction (cycloversion) versus those in which the eyes move in opposite directions (cyclovergence). They found that the variability in cycloversion is much larger than in cyclovergence; the torsional component of movements during fixation is generally conjugate. However, these studies did not particularly analyze the torsional component of microsaccades, focusing instead on the overall pattern of stability during fixation, which is dominated by drift. A recent study used video eye-tracking to measure the torsional component of microsaccades in a single subject (11), but a detailed characterization of the torsional component of microsaccades is still lacking.

Torsional optokinetic stimuli produce a torsional nystagmus (12). During optokinetic nystagmus, eye movements alternate between slow phases towards the direction of the rotating stimulus and quick phases in the opposite direction. Quick phases of nystagmus are practically indistinguishable from saccades (13) and are partially generated by the same pre-motor circuitry. Thus, our analyses will combine all saccadic-like eye movements during fixation, including microsaccades and quick phases of nystagmus.

Here we take advantage of a newly developed method to record eye movements around the three axes of rotation at high speeds and in real time (14) to characterize the torsional component of microsaccades during fixation. Moreover, we compare the movements during fixation at a stationary stimulus with those during fixation in the presence of a torsional optokinetic stimulus. We will use the term microsaccade in the context of fixation on a stationary stimulus and the term quick phase in the context of a rotating stimulus.

## Methods

### Subjects

Seven right-handed subjects (mean age 28.3, 23-35 years old, five females, two males) participated in our study. All participants were healthy and had no history of neurological disorders, and did not take medications known to interfere with eye movements. Participants did not wear glasses during the experiment to optimize the quality of the eye movement recordings, avoiding reflections, distortions, or occlusions caused by the lenses or the glasses' frames. All subjects could fixate on the central target. Two subjects had myopia, and one had a history of myopia corrected by photorefractive keratotomy. One of the myopic subjects wore soft contact lenses during the experiment, but they were not an impediment to good recordings during central fixation. The experimental protocol was approved by the Johns Hopkins institutional review board, and written informed consent was obtained from all participants. Subjects were reimbursed for their participation.

### Apparatus

Subjects sat in front of an OLED 55-inch 4K ultra-HD monitor screen (OLED55B7P-U, LG, Seoul, South Korea) and 60 cm away from it, with the center of the visual stimulus in the center of their visual field. The screen was 121 cm wide and 68 cm tall, providing a horizontal visual field of 90 degrees and a vertical visual field of 60 degrees. To reduce head motion, we used a bite bar molded for each subject.

**Figure 2.**
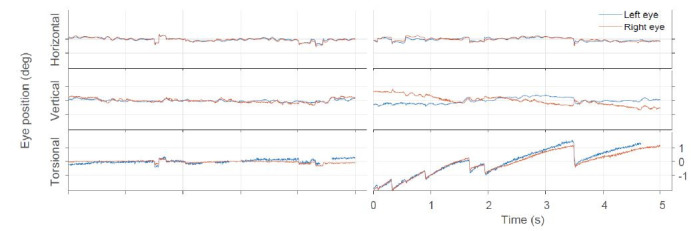
Example traces for the three components of eye movements around each axis of rotation for fixation of stationary stimulus (left) and optokinetic stimulus (right).

A custom-made eye tracker was used to record eye movements. The eye tracker was composed of a camera (Flir GS3-U3-23S6M-C), lens (Fujinon CF35HA1), infrared illuminator (CMVision IR30 WideAngle IR Illuminator), and infrared filter (ZOMEi 49MM IR 720). The software controlling the camera and recording the eye movements was a modified version of the software previously described for a goggle system by Otero-Millan and colleagues (14). This method calculates torsion using template matching in real-time of the iris pattern with a reference iris pattern acquired during initial calibration. Before template matching, the iris pattern is transformed from a ring into a rectangle, so rotations become translations and optimized with a bandpass filter to enhance the iris features and account for changes in luminance. Recordings and measurements can be obtained binocularly with a noise level of less than 0.1° and around the three axes of rotation. The noise level can be higher for eccentric positions and for eyes with less salient iris pattern. In the updated version, the camera's capabilities made it possible to record eye movements at 250Hz, as opposed to 100 Hz (14). Recordings were calibrated using a behavioral five-point calibration. Figure 2 shows an example of eye position traces for stationary conditions and clockwise rotation at 30 deg/s.

### Experimental paradigm and visual stimuli

Each experiment consisted of an approximately one-hour session for each subject. During the session, subjects were presented with 64 trials, each lasting 30 seconds. The visual stimulus in all trials included a red fixation spot of 0.5 deg diameter and a random pattern of dots of various sizes that rotated around the fixation target at different speeds (Figure 1B). The stimulus rotated around the fixation spot at a constant speed during each trial selected among the following values: 30 deg/s, 20 deg/s, 10 deg/s, or 0 deg/s (no rotation) in the clockwise or counterclockwise directions, and subjects repeated each condition eight times. Conditions were randomly interleaved, and at the end of each trial, the subjects had the option of pressing a key for the next trial to begin; therefore they had control over the pace and could take short breaks in between trials. The stimulus was composed of 2000 solid white circles (dots) over a black background. The solid circles of random sizes (from 1 to 10 deg diameter) were placed randomly within a ring no closer than 1 deg to and no further than 40 deg from the fixation target. Stimuli were generated with the Psychophysics Toolbox (15).

### Data analysis

Microsaccades and quick phases of nystagmus are practically indistinguishable. Thus, we used a common method to detect all saccade-like movements during our experiments. We used a modified version of the method developed by Engbert and Kliegl (16) adapted to also detect the torsional component. That is, instead of using an ellipse in the plane of horizontal and vertical velocities as the threshold, we calculated an ellipsoid that acted in the same manner in the three dimensions. We used a factor of six to determine the threshold by multiplying by that factor the standard deviation of the velocity in our data.

Some studies restrict microsaccades to those detected simultaneously in both eyes. However, having clear recordings from only one eye can be a common occurrence in torsional recordings given the difficulty of tracking the iris. Here we included in the analysis saccades that were detected in both eyes and saccades that were detected only in one eye but had velocities three times larger than the velocity threshold calculated to detect potential saccades in each of the components.

Before running the detection algorithm, we preprocessed the data to remove portions of eye movement recordings that included blinks, eye tracking failures, or artifacts. First, we used the pupil size data to identify portions of data where the pupil size changed too fast. We identified samples that were the pupil was 10 pixels (around 1mm) larger or smaller with respect to a robust estimate of a smoothed pupil size trace, or where the pupil size changed by more than 10000 pixels/s. Then, we identified portions with unnatural velocities (more than 1000 deg/s) and/or accelerations (more than 50000 deg/s2) in the horizontal and vertical components. Subsequently, we removed the portions of data identified as artifacts or blinks from all three components with an additional 100ms before and after. Finally, we repeated the process only for the torsional component so as not to remove data from the horizontal and vertical components unnecessarily with a threshold for the velocity of (500 deg/s) and for the acceleration of (20000 deg/s2). Thus, some sections of the data may have missing torsion data while still having horizontal or vertical data.

For the statistical analysis of fixation, we first fit a regression line to estimate the slopes for each subject. Then we used a paired t-test to compare the slopes for different components. For the statistical analysis of the relationship between quick phase parameters and stimulus velocity or between the different components, we used a linear mixed model with random intercept and slope and reported the significance of the fixed slope effect.

## Results

**Figure 3.**
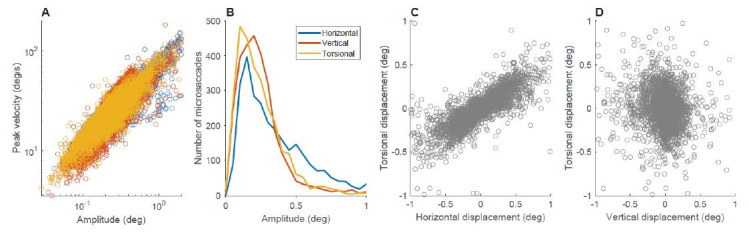
Microsaccade characteristics during fixation. A) Main sequence of the three components of microsaccades. B) Amplitude distributions of microsaccades. C) Relationship between the horizontal and torsional displacement during each microsaccade. D) Relationship between the vertical and torsional displacement during each microsaccade. Data from all conditions with zero velocity of rotation and all subjects combined.

During fixation with the stationary background, microsaccades occurred at an average rate of 1.0 ± 0.2 (mean±sem) microsaccades/s. The average amplitude for each of the components was 0.5 ± 0.1 deg for the horizontal, 0.25 ± 0.04 deg for the vertical, and 0.34 ± 0.07 deg for the torsional component. Figure 3 shows a summary of the characteristics of the horizontal, vertical, and torsional components of microsaccades. Figure 3A shows the relationship between amplitude and peak velocity, sometimes referred to as *the main sequence*. The corresponding average R-squared values were 0.69, 0.6, and 0.76 for the horizontal, vertical, and torsional components, respectively. None of the pairwise comparisons were significantly different, indicating that for small saccade sizes all three component appear to follow the same relationship (p>0.1 in all cases). The amplitude distributions in Figure 3B show more occurrences of microsaccades above 0.5 deg in the horizontal component. Figures 3C shows that the horizontal and torsional components of the displacement during a microsaccade are correlated with each other (t (6) =4.5, p=0.004), while Figure 3D shows that there is no relationship between the vertical and torsional components (t (6) =-1.8, p=0.27).

**Figure 4.**
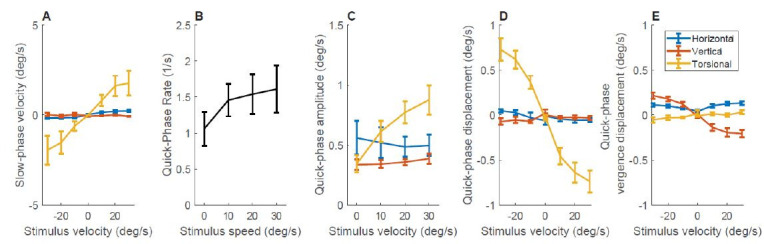
The effect of stimulus velocity on eye movements. A) The effect of stimulus velocity on slow-phase velocity. B) The effect of stimulus velocity on quick-phase rate. Positive and negative velocities were combined because of symmetry. C) The effect of stimulus velocity on the amplitude of each component of quick phases. Positive and negative velocities were collapsed because of symmetry. D) The effect of stimulus velocity on the displacement of each component of quick phases. We define displacement as the difference between the start position and the end position. Amplitude is defined as the distance between the two farthest points during the movement for each of the components. E) The effect of stimulus velocity on quick-phase vergence components. Here we subtracted the displacement of the left eye from the displacement of the right eye. Note that the data at zero velocity corresponds with the data shown in Figure 3.

The rotating stimulus, as expected, induced a mostly torsional optokinetic nystagmus characterized by its slow-phase velocity (Figure4A). The average velocity of the slow phases correlated with the rotational velocity of the stimulus in the horizontal and torsional components (t(54)=6.06, p<0.0001 and t(54)=2.8, p=0.006 respectively but not in the vertical component (t(54)=-0.6, p=0.53). For quick phases, we measured their rate as the number of occurrences per second (Figure 4B); their amplitude as the distance between the two farthest points during the movement for each of the component (Figure 4C); their displacement as the signed change in eye position from the beginning to the end of the movement (Figure 4D); and their vergence displacement as the signed change in vergence eye position from the beginning to the end of the movement (Figure 4E) and. The rate of quick phases showed a trend to increase with higher stimulation velocities (t (26) =1.8, p=0.08), consistent with the need for more-frequent position corrections due to the faster slow-phase velocity speed. The amplitude of the torsional component of the quick phases increased with speed as well (t (26) =6.8, p<0.0001). The displacement of the torsional component of the quick phases correlated with the rotational velocity of the stimulus (t (54) =-6.7, p<0.0001). However, when looking at vergence, the vertical component also showed a clear correlation with rotational speed (t (54) =-6.6, p<0.0001). This is consistent with the see-saw nystagmus pattern typical of stimulation in the roll plane where one eye moves slightly up while the other one moves down and both simultaneously rotate torsionally in the same direction.

## Discussion

Technologies that made it straightforward to measure eye movements around three axes of rotation have been scarce. This has limited the number of studies investigating the torsional component of fixational eye movements. Previous studies have addressed the general patterns of instability during fixation in three dimensions, but little is known about the particular properties of the torsional component of microsaccades. Here, we measured eye movements binocularly with new software that can achieve 250Hz recordings. The newly developed software and hardware is capable of measuring small eye movements, including torsion, to characterize microsaccades around three axes of rotation. Our results are compatible with the examples of microsaccades shown in a previous study (11), but we expand further by showing a more detailed description of a larger set of microsaccades from multiple subjects.

First, we described the parameters of the torsional component of microsaccades during fixation: they are on average of similar amplitude (0.34 degrees) as the horizontal component (0.5 degrees). The amplitude of the torsional component is larger than what would be expected simply from crosstalk between horizontal/vertical and torsional components, suggesting a true independent torsional component appearing during fixation.

We have also shown that the displacement of quick phases in the vertical and torsional components is correlated with the velocity of the rotational stimulus. The horizontal component, however, appears to be independent of the stimulus velocity. This is consistent with the fact that the torsional and vertical components are generated in the same brain stem nuclei (rostral interstitial nucleus of the medial longitudinal fasciculus, riMLF) while the horizontal component is generated by a set of different nuclei (paramedian pontine reticular formation, PPRF) (17).

The circuits that generate saccades and microsaccades have been extensively studied. However, less is known about the circuits that produce quick phases of nystagmus. Recent models of the generation of microsaccades have proposed that at a neural level, a trigger mechanism where fluctuations inactivity in the superior colliculus (SC) switch the state of a mutually inhibitory circuit in the brain stem formed by the omnipause neurons (OPNs) and the burst neurons (BNs) (18). This is not however, confirmed to be the case at a behavioral level (19). The triggering mechanism for quick phases of nystagmus is not clearly understood. It may involve the same OPNs and BNs as for saccades as well as a different type of neurons (burster driver neurons) in the brain stem (20). It is unclear if the SC plays any role in generating quick phases of nystagmus.

### Ethics and Conflict of Interest

The authors declare that the contents of the article are in agreement with the ethics described in http://biblio.unibe.ch/portale/elibrary/BOP/jemr/ethics.html and that there is no conflict of interest regarding the publication of this paper.

### Acknowledgements

We wish to thank Dale Roberts for technical assistance, and Jing Tian, David S. Zee and Amir Kheradmand for their comments. This work was supported by the National Eye Institute (Award K99EY027846 to JOM).
